# Regulatory Mechanism of the GmMYB14 Transcription Factor on Auxin-Related Proteins in Soybean

**DOI:** 10.3390/ijms26167763

**Published:** 2025-08-11

**Authors:** Lihua Peng, Yangyan Liu, Hongli Yang, Wei Guo, Qingnan Hao, Shuilian Chen, Songli Yuan, Chanjuan Zhang, Zhonglu Yang, Bei Han, Yi Huang, Zhihui Shan, Limiao Chen, Haifeng Chen

**Affiliations:** 1 Key Laboratory of Biology and Genetic Improvement of Oil Crops, Ministry of Agriculture and Rural Affairs, Oil Crops Research Institute of Chinese Academy of Agricultural Sciences, Wuhan 430062, China; penglihua2025@163.com (L.P.); lyy08050525@163.com (Y.L.); yanghongli@caas.cn (H.Y.); guowei03@caas.cn (W.G.); haoqingnan@caas.cn (Q.H.); chenshuilian@caas.cn (S.C.); yuansongli@caas.cn (S.Y.); zhangchanjuan@caas.cn (C.Z.); yangzhonglu@caas.cn (Z.Y.); hanbei@caas.cn (B.H.); huangyi@caas.cn (Y.H.); shanzhihui@caas.cn (Z.S.); 2 College of Agriculture, Yangtze University, Jingzhou 434000, China

**Keywords:** soybean, plant architecture, MYB transcription factor, indole-3-acetic acid (IAA), *GmIAA1*

## Abstract

In a previous study, *GmMYB14* overexpressing (*GmMYB14-OX*) transgenic soybean plants displayed a semi-dwarfism and compact phenotype, which was regulated by the brassinosteroid (BR) pathway. However, the phenotype of *GmMYB14-OX* plants could be partly rescued after spraying them with exogenous BR. This indicates that other hormones, in addition to BR, also play a role in regulating the architecture of *GmMYB14-OX* plants. We observed a significant decrease in the content of endogenous indole-3-acetic acid (IAA) in transgenic soybean lines (OX9 and OX12) compared to wild type (WT) plants. The plant height, leaf area, leaf petiole length, and leaf petiole angle of *GmMYB14-OX* plants could also be partly rescued after applying exogenous IAA for two weeks. Transcriptome sequencing analysis revealed that the expression of many genes within the Aux/IAA gene family underwent alterations in the *GmMYB14-OX* transgenic soybean plants. Among them, *Glyma.02G000500* (*GmIAA1*) showed the highest expression in *GmMYB14-OX* plants. Furthermore, the results of electrophoretic mobility shift assay and dual-luciferase reporter indicate that GmMYB14 protein could bind to the promoter of *GmIAA1*. In summary, a decrease in endogenous IAA content may be one of the factors contributing to the compact and dwarfed architecture of *GmMYB14-OX* plants. GmMYB14 also acts as a transcriptional activator of *GmIAA1* to potentially block IAA effects. Our findings provide a theoretical basis for further investigation of the regulatory mechanism of GmMYB14 on soybean plant architecture.

## 1. Introduction

Soybean is a crucial oil and forage crop, boasting abundant plant proteins and oils [1]. It serves as a significant source for various agricultural products [2], health supplements [1,3], and animal feed [4]. With the continuous growth of the world’s population, the global demand for soybeans is also increasing.

As a podding crop, the yield of soybean is affected by plant architecture, including plant height, stem diameter, node number, internode length, branch number, leaf petiole length, leaf petiole angle, leaf size, etc. [5,6]. Unlike wild soybeans, cultivated soybeans have undergone multiple rounds of domestication. They generally exhibit an erect stem growth habit, with thicker stems, larger inflorescences, and bigger seeds compared to wild soybeans [5,7]. Over the past few decades, semi-dwarf breeding has been achieved in crops such as rice and wheat. Semi-dwarf varieties possess stronger lodging resistance and higher yields, which has inspired breeders to enhance the lodging resistance and yield of soybeans by selecting and breeding semi-dwarf varieties [8]. The petiole angle directly affects the canopy structure of crops. A smaller petiole angle results in a more compact plant, which not only influences light interception and photosynthetic efficiency but also effectively increases the planting density of crops and land use efficiency, thereby leading to an increase in crop yield per unit area [8,9].

MYB transcription factors (TFs) constitute one of the largest TFs families in plants. Previous research has revealed that MYB TFs not only play a role in regulating the synthesis of flavonoid secondary metabolites and responding to abiotic stresses, such as drought and cold, but also participate in the regulation of plant architecture [10,11,12]. MYB110 was a Pi-dependent negative regulator of plant height. Under both low phosphorus and high phosphorus conditions, the *myb110* mutant exhibited increased rice plant height and enhanced lodging resistance [13]. Li et al. found that Dissected Leaf 1 (StDL1), an R2R3 MYB TF, regulated the dissected leaf morphology in potato (*Solanum tuberosum* L.), and knocking out *StDL1* resulted in the non-dissected leaf morphology in plants [14]. Notably, some MYB TFs regulate the architecture of higher plant height through several hormonal pathways. Han et al. discovered that FveMYB117a inhibits the number of axillary buds during crown outgrowth in woodland strawberry (*Fragaria vesca* L.) by negatively regulating cytokinin (CK) synthesis [15]. In *Oryza sativa*, overexpression of *OsMYB14* negatively regulated the expression of genes related to gibberellin (GA) synthesis, leading to reduced plant height [16]. In *Arabidopsis*, the *AtMYB30* knockout mutant showed decreased BR levels and dwarfed plant growth [17]. In our previous study, overexpression of the *GmMYB14* inhibited BR synthesis, resulting in reduced soybean plant height, decreased leaf area, and smaller petiole angles and lengths [12].

Indole-3-acetic acid (IAA) is a crucial class of plant hormones that play significant roles in plant growth, development, and morphogenesis. The auxin/indole-3-acetic acid (Aux/IAAs) gene family represents a group of early auxin responsive genes that act as repressors in the classical auxin signaling pathway [18]. Under low levels, Aux/IAA proteins can form dimers with auxin response factors (ARFs) through their PB1 domains, thereby impeding the interaction between ARFs and auxin response elements (AuxREs). Under high levels, auxin, Aux/IAA proteins, and the auxin receptor TRANSPORT INHIBITOR RESPONSE 1 (TIR1)/AUXIN SIGNALING F-BOX (AFB) form a ternary complex, which is subsequently recognized and ubiquitinated by the SKP1-CUL1-F-BOX (SCF)-type ubiquitin–protein ligase complex. Eventually, the Aux/IAA proteins are degraded by the 26S proteasome, relieving the repression on transcription [19,20]. It has been reported that Aux/IAA proteins are involved in lateral root development, aerenchyma formation, promotion of floral organ development and flowering, regulation of apical hook formation, and responses to abiotic stresses, such as salt and drought [21,22,23,24,25].

Some research has revealed that the functions of MYB TFs are associated with Aux/IAA proteins. In *Brassica napus* L., silencing of *BnMYB69* led to reduced plant height, which was associated with impaired IAA synthesis. After supplementation with exogenous IAA, the dwarf phenotype was largely restored [26]. Overexpression of *GmMYB133* in soybean led to shortened hypocotyls, reduced plant height, increased stem diameter, and significantly decreased endogenous free IAA and GA contents [27]. Yuan et al. found that under low auxin concentrations, the development of tomato trichomes was negatively regulated by the SlARF4-SlTHM1/SlMYB52-SlIAA15 network. At higher auxin concentrations, SlIAA15 was degraded, releasing SlARF4, which then inhibited the expression of *SlTHM1*/*SlMYB52*, thereby alleviating the suppression of trichome development [28]. In *Camellia sinensis* (L.), the interaction between CsMYB4a and CsIAA4 impeded the degradation of CsIAA4 and inhibited filament growth [29]. However, the role of MYB TFs in conjunction with Aux/IAA proteins in regulating plant architecture remains poorly understood.

In our previous study, overexpression of *GmMYB14* inhibited BR synthesis, resulting in reduced plant height, decreased leaf area, and smaller petiole angles and lengths in soybean plants [12]. After application of exogenous BR, the plant architecture was partially restored to normal levels, suggesting that other hormones were also involved in regulating the compact plant architecture. In this study, we discovered that many genes related to IAA synthesis and transport were up regulated in *GmMYB14-OX* plants, in which the endogenous IAA content was significantly decreased. Gel shift assays and dual-luciferase reporter assays demonstrated that *GmIAA1* was one of the downstream target genes of GmMYB14. After supplementation with exogenous IAA, the plant architecture of the *GmMYB14-OX* plants could be largely restored, proving that the dwarfed and compact plant architecture of the *GmMYB14-*OX lines is associated with reductions in endogenous IAA levels.

## 2. Results

### 2.1. Endogenous IAA Content of GmMYB14-OX Plants Decreased

**Figure 1:**
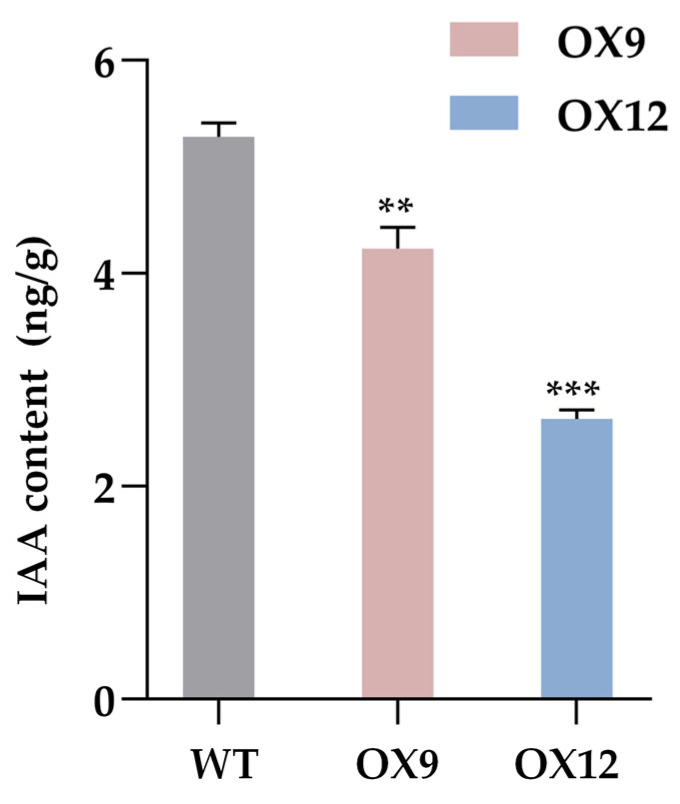
Endogenous IAA content of *GmMYB14-OX* and WT plants. Data are shown with three biological replicates. The values are the means ± SD (*n* = 3). Statistically significant differences between OX9 or OX12 and WT are marked with asterisks (** *p* < 0.01, and *** *p* < 0.001; Student’s *t*-test).

We hypothesized that auxin may regulate the semi-dwarf and compact phenotype of transgenic *GmMYB14-OX* plants, in addition to BR [12]. Therefore, we measured the endogenous IAA content of OX9 and OX12 lines and WT plants. The results show that compared with WT, the endogenous IAA content of both OX9 and OX12 were significantly reduced (Figure 1), suggesting that the semi-dwarf and compact phenotype of transgenic *GmMYB14-OX* plants may be associated with reducing endogenous IAA levels.

### 2.2. Phenotype of GmMYB14-OX Plants Partially Recovered After Treatment with IAA

We explored whether spraying exogenous IAA on OX9 and OX12 plants could rescue the semi-dwarf phenotype, which may provide direct evidence for the semi-dwarfism associated with the GmMYB14 mediated decrease in IAA levels in transgenic soybean plants.

**Figure 2:**
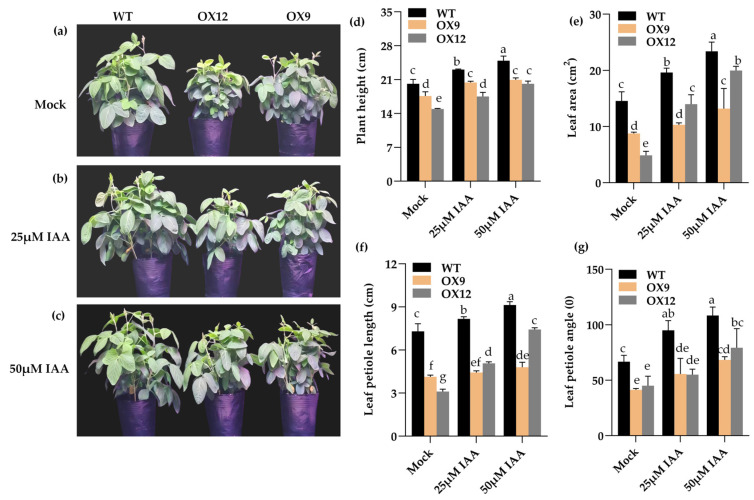
Exogenous IAA regulated the plant architecture of transgenic *GmMYB14-OX* plants and WT plants. (**a**–**c**) Phenotypes of OX9, OX12, and WT plants after treatment with 0, 25, and 50 μM of IAA for two weeks. (**d**–**g**) Plant height, leaf area, leaf petiole length, and leaf petiole angle of the OX9, OX12, and WT plants after treatment with 0, 25, and 50 μM IAA for two weeks. Data are shown with 3 biological replicates. The values were the means ± SD (*n* = 3). Significant differences are shown with different letters following Duncan’s multiple range test (*p* < 0.05).

We sprayed 25 and 50 μM of IAA or mock treatment on the leaves of OX9, OX12, and WT plants. After two weeks of treatment, we measured the plant height, leaf area, leaf petiole length, and leaf petiole angle of the OX9, OX12, and WT plants. The results show that the plant height, leave area, leaf petiole length, and leaf petiole angle of the OX9, OX12, and WT plants were partially recovered (Figure 2), while the phenotype of the control plants showed no significant change. These results provide evidence that the semi-dwarf and compact phenotype of *GmMYB14-OX* plants is associated with endogenous IAA levels decreasing.

### 2.3. Differentially Expressed Genes (DEGs) in GmMYB14-OX and WT Plants

The leaves of WT and homozygous T3 generation OX9 plants were collected for RNA-seq analysis at day 24 after sowing (24 DAS). After filtering the raw data obtained from Illumina HiSeq sequencing (BGI Genomics, Shenzhen, China), the clean reads acquired for the WT plants and OX9 line were 40.8 M and 40.5 M, respectively, with a read length of 150 bp. The Q30 rate (%) and GC content (%) of the WT and OX9 line were 93.1% and 45.0%, 93.8%, and 45.1%, respectively. Subsequently, the clean reads were aligned to the reference genome using HISAT software (version: v2.0.4), with total mapping of 84.13% and 84.56% for the WT plants and OX9 line, respectively [30]. The link for the reference genome is https://phytozome-next.jgi.doe.gov/info/Gmax_Wm82_a4_v1 (accessed on 25 April 2024). DEG detection was performed using DEseq2 software (version: v1.10.1), as described by Love et al. [31].

The results show that 2923 DEGs (1648 up regulated and 1275 down regulated) were identified in leaves [12]. Among them, 25 auxin related genes were identified. We found several auxin related genes that were up regulated, including *G. Max* AUXIN/INDOLE-3-ACETIC ACID (*GmIAA*, *Glyma.02G000500* and *Glyma.05G229300*); *G. Max* AUXIN RESPONSE FACTORS (*GmARF*, *Glyma.14G217700* and *Glyma.10G210600*); *G. Max* AUXIN BINDING PROTEINS (*GmABP*, *Glyma.15G176900* and *Glyma.02G150100*); *G. Max* AUXIN RESISTANT 1/LIKE AUXIN RESISTANT (*GmAUX1*/*GmLAX*, *Glyma.02G255800*); *G. Max* AUXIN RESISTANT (*GmAXR4*, *Glyma.03G237400*). Meanwhile, *G. Max* AUXIN RESISTANT 1/LIKE AUXIN RESISTANT (*GmAUX1*/*GmLAX*, *Glyma.06G110200* and *Glyma.03G063600*); *G. Max* PIN FORMED PROTEIN (*GmPIN*, *Glyma.18G241000* and *Glyma.07G164600*); *G. Max* SMALL AUXIN UP RNA (*GmSAUR*, *Glyma.08G004100*, *Glyma.09G221900*, *Glyma.09G221800* and *Glyma.16G020700*) and *G. Max* IAA LEU RESISTANT 1 (*GmILR1*, *Glyma.06G115100*) were down regulated in OX9 leaves (Table 1).

### 2.4. High Expression of GmIAA1 in OX12 Leaves

**Figure 3:**
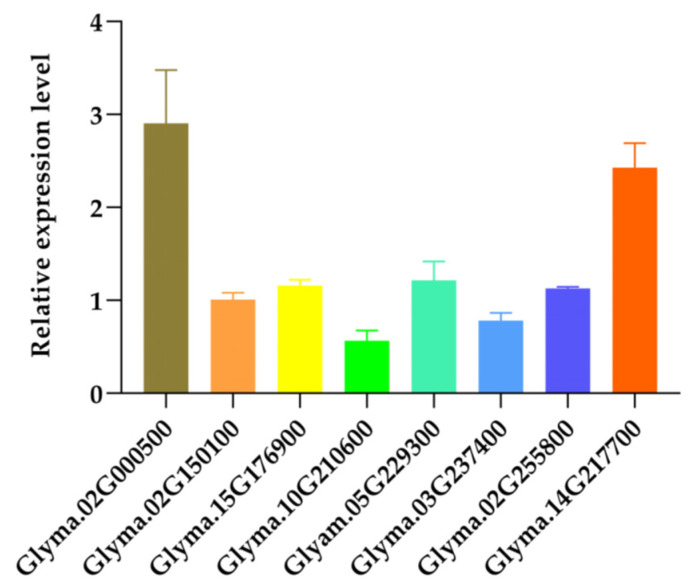
Relative expression levels of auxin related genes in OX12 leaves. Data are shown with 3 biological replicates. Relative expression levels were normalized to the expression levels of Tianlong No. 1 (WT).

GmMYB14 acts as a transcriptional activator, as demonstrated by a transactivation activity assay in a previous study [12]. To find the target gene of GmMYB14 in the auxin pathway, the expression levels of 8 genes upregulated in transcriptome analysis were verified by qRT-PCR. The results show that *Glyma.02G000500* (*GmIAA1*), *Glyma.15G176900*, *Glyma.05G229300*, and *Glyma.14G217700* were up regulated in OX12 leaves, which is basically consistent with the transcriptome results (Figure 3). Furthermore, we found that *GmIAA1* exhibited the highest up regulation (2.9 fold) compared to WT. Therefore, we speculate that *GmIAA1* may be a direct target gene of GmMYB14 in the auxin signaling pathway.

### 2.5. GmMYB14 Directly Regulated the Expression of GmIAA1

**Figure 4:**
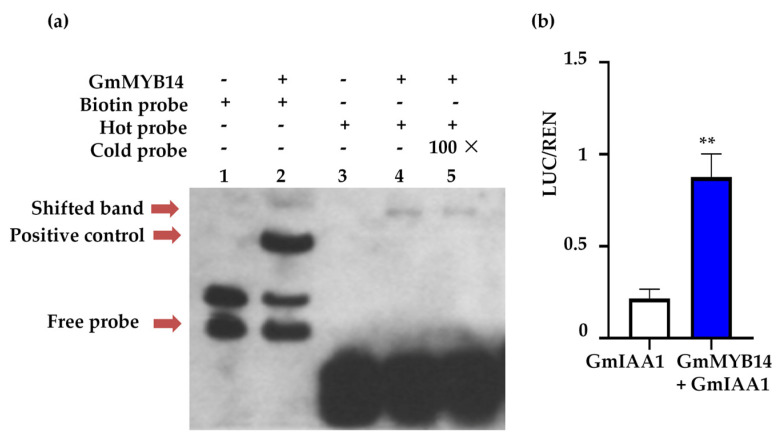
GmMYB14 TF activates *GmIAA1* expression. (**a**) Electrophoretic mobility shift assay (EMSA) showing the interaction between GmMYB14 and *GmIAA1*. *GmIAA1* was used as the hot probe, with a 100 × competitive cold probe used for the binding competition test. Lanes 1, 3: Only included biotin probe or hot probe, used as negative controls. Lane 2: Co incubation of GmMYB14 protein and biotin probe as positive controls. Lane 4: Co-incubation of GmMYB14 protein and hot probe (shifted band). Lane 5: Interaction between GmMYB14 protein and hot probe, with the competition test by a 100 × cold probe. (**b**) GmMYB14 protein enhanced the transcription activity of LUCIFERASE (LUC) reporter gene driven by pGmIAA1 promoter in *Arabidopsis* protoplasts. LUC activity was normalized to the respective Renilla luciferase (REN) activity and expressed as relative luminescence units. Data are presented as mean ± SD (*n* = 3). Statistical significance was determined by Student’s *t*-test (** *p* < 0.01).

To investigate whether GmMYB14 directly regulated the expression of *GmIAA1*, our study used *GmIAA1* as a hot probe and GmMYB14 protein as the target protein in an electrophoretic mobility shift assay (EMSA). The results show that when both GmMYB14 protein and the hot probe were present, shifted bands appeared on the gel. However, when cold probe was added, the color of the shifted band slightly faded (Figure 4a). Subsequently, we performed a transactivation assay in *Arabidopsis* protoplasts using the pGmIAA1::*LUC* reporter plasmid to verify whether GmMYB14 directly binds to the promoter region of *GmIAA1*. The results show that compared with the control, the promoter activity was significantly enhanced upon GmMYB14 binding to *GmIAA1* (Figure 4b), demonstrating that GmMYB14 directly binds to the *GmIAA1* promoter region and regulates its expression.

## 3. Discussion

The MYB transcription factor is one of the largest transcription factor families in plants. It not only participates in the synthesis of plant secondary metabolites and responds to various abiotic stresses, but it also regulates the plant architecture of multiple species, such as soybean [12], rice [16], and strawberry [15]. Previous research identified a transcription factor that negatively regulates the biosynthesis and accumulation of BR, thereby mediating a semi-dwarfism and compact plant architecture in *GmMYB14-OX* plants. However, when exogenous BR was supplemented, the plant architecture of *GmMYB14-OX* did not fully revert to the level of the wild type plants. Therefore, we hypothesized that the plant architecture of the *GmMYB14-OX* lines was also regulated by other hormones.

It is well known that auxin is the first discovered plant hormone, and the morphological development of plants depends on auxin regulation. The steady state level of auxin in cells is not only related to the metabolism of auxin but also affected by the transmembrane transport of auxin [32,33]. Some studies have found that plants can mediate the asymmetric distribution of auxin levels in cells through polar transport, thereby regulating the development of organs, such as stem meristem and leaf primordia formation and lateral root development [34,35,36,37]. In *Oryza sativa*, OsMYB7 increased leaf angle by reducing auxin accumulation and promoted cell elongation at the adaxial side of lamina joints [38]. Zhang et al. found that OsPIN1b regulated the rice leaf angle through the IAA pathway. In *OE-OsPIN1b-1* plants, the free auxin content in the lamina joint was significantly reduced, while adaxial cell division in the pulvinus increased, ultimately promoting leaf inclination [39]. In soybean, the transcripts of *GmPIN1* exhibited an asymmetric distribution at the base of the petiole. The lower cells of the petiole showed higher expression levels of *GmPIN1* and elevated auxin concentrations, leading to asymmetric expansion of cells at the petiole base and resulting in changes in the petiole angle of the plant [40]. Additionally, GmPIN1 participated in nodule primordium formation through polar auxin transport [41].

In this study, we measured endogenous auxin levels in two *GmMYB14-OX* transgenic lines (OX9 and OX12) and found that both lines exhibited significantly reduced auxin levels compared to the control (Figure 1). These results suggest that the observed phenotypes in *GmMYB14-OX* plants may be associated with decreased endogenous auxin content. Interestingly, several auxin influx carrier *GmAUX1s*/*GmLAXs* (*Glyma.03G063600* and *Glyma.06G110200*), auxin efflux carrier *GmPINs* (*Glyma.07G164600* and *Glyma.18G241000*) were down regulated in OX9 leaves. *AtAUX1*, a homologous gene of *Glyma.03G063600*, regulated the accumulation of auxin in callus tissue, and the auxin levels in the *aux1-22* mutant callus were significantly reduced [42,43]. *AtLAX3*, the homologous gene of *Glyma.06G110200*, has been demonstrated to alter intracellular auxin levels by regulating auxin influx activity [44,45]. These results suggest that both *Glyma.03G063600* and *Glyma.06G110200* may play roles in modulating auxin levels in soybean. Furthermore, although the functions of the soybean PIN family members *Glyma.07G164600* and *Glyma.18G241000* remain uncharacterized, their homologous genes *AtPIN3* and *AtPIN5* are known to participate in the regulation of intracellular auxin homeostasis, implying that *Glyma.07G164600* and *Glyma.18G241000* may possess important biological functions [46,47,48]. IAA amino acid hydrolase ILR1 like 6 (*Glyma.06G115100*), a member of the soybean *ILR* gene family, down regulated in *GmMYB14-OX* lines, may be affected the auxin content of the *GmMYB14-OX* lines by converting amino acid conjugated auxin into free auxin [33,49].

On the other hand, auxin signaling transduction also played critical roles in plant growth and development. Several auxin signaling related genes, such as *OsARF4* and *OsARF19*, regulated the rice leaf angle by modulating asymmetric cell elongation or division [50,51]. Shin et al. demonstrated that the interaction between AtMYB77 and AtARF7 significantly reduced the lateral root number in *Arabidopsis* [52]. GmMYB81, the soybean homolog of AtMYB77, was also predicted to interact with ARFs to regulate plant development [53]. Song et al. revealed that OsIAA1 interacted with OsARF1, and *OsIAA1* overexpressing plants exhibited morphological changes, including dwarfism and increasing leaf angles [54]. Similarly, the GmIAA27 protein mediated dwarf and multi-branching phenotypes in soybean, where amino acid mutations in its coding region disrupted its binding to *GmTIR1* [55]. In this study, we found that *GmIAA1* displayed the most significant up regulation in the leaves of *GmMYB14-OX* plants (Figure 3), further confirming an interaction between GmMYB14 and *GmIAA1* (Figure 4). Ma et al. reported that the interaction between CsMYB4a and CsIAA4 blocked the ubiquitination degradation of CsIAA4 induced by α-NAA, thereby inhibiting filament elongation in tobacco [29]. We hypothesize that the binding of the GmMYB14 protein to *GmIAA* may affect the stability of the GmIAA protein, and this hypothesis will be verified in future experiments. However, in this study, three genes in the *AUX/IAA* subfamily (*Glyma.20G210500*, *Glyma.02G142500*, and *Glyma.10G031900*) expression were down regulated. Among these, *Glyma.20G210500* is the homologous gene of *GmIAA1*. The down regulation of these genes suggests the presence of a compensatory mechanism, which results in a less pronounced decline in the endogenous content of IAA.

Notably, foliar application of either 25 μM or 50 μM IAA increased plant height, leaf area, leaf petiole angle, and leaf petiole length in *GmMYB14-OX* transgenic lines, although these morphological parameters failed to fully recover to wild type levels (Figure 2). Since spraying exogenous IAA or BR alone can partially restore the phenotype of the *GmMYB14-OX* lines, it is highly necessary to further investigate whether co-spraying exogenous IAA and BR can fully restore its phenotype [12]. In fact, we are currently exploring the optimal concentrations of IAA and BR in a mixed solution to avoid inhibiting plant growth due to excessively high hormone concentrations, which could compromise the accuracy of the experiment.

Transcriptome analysis revealed that DEGs in OX9 leaves were significantly enriched in the environmental adaption, lipid metabolism, biosynthesis of secondary metabolites, amino acid metabolism, and signal transduction pathways [12]. These DEGs may be involved in growth and morphological development. Moreover, recent reports have shown that MYB TFs regulated plant height, leaf shape, and axillary bud growth through the GA and CK pathway, respectively [15,17,56]. For other approaches, further verification through experiments is needed.

## 4. Materials and Methods

### 4.1. Plants Materials

In a previous study, the CDS of *GmMYB14* was cloned into the pB2GW7 expression vector under the control of the cauliflower mosaic virus CaMV *35S* promoter, and the construct was then transferred into the *Agrobacterium tumefaciens* EHA105 to transform the Tianlong No. 1 cultivar. The stable *GmMYB14-OX* lines OX9 and OX12 have been verified to contain a single copy of the transgene, as evidenced by Southern blot analysis [12]. Here, *GmMYB14-OX* homozygous lines (OX9, OX12) were used as plant materials, and they all displayed a semi-dwarfism and compact phenotype. The plump, uniformly sized, and disease free seeds of the OX9, OX12, and Tianlong No.1 (WT) were cultivated in pots (height × top diameter = 18.5 × 18.5 cm) filled with a mixture of vermiculite and nutrient soil (vermiculite:nutrient soil = 1:2). After the unifoliolate leaves had unfolded, each pot retained four seedlings. Three biological replicates per line were used in the analysis. The seedlings were grown in a greenhouse under controlled conditions of 28 °C with a 16 h light/8 h dark photoperiod.

### 4.2. Endogenous IAA Content Measurement

Soybean plants were grown in pots (height × top diameter = 18.5 × 18.5 cm) with two plants per pot in a greenhouse under 16 h light/8 h dark photoperiod conditions at 28 °C. Shoot parts were harvested from two week old seedlings. Three biological replicates per line were used in the analysis. Endogenous free IAA contents were quantified using ultra performance liquid chromatography electrospray ionization–tandem mass spectrometry (UPLC-ESI-MS/MS), as previously described by Lee et al. [57]. The endogenous IAA from the shoots of OX9, OX12, and WT plants was extracted using an isopropanol/water/hydrochloric acid extraction method. The endogenous IAA was then quantified using an Agilent 1260 high performance liquid chromatography (HPLC) system (Agilent Technologies, Santa Clara, CA, USA) coupled with an AB Qtrap6500 (AB Sciex, Framingham, MA, USA) mass spectrometer. The IAA standard sample was purchased from Sigma-Aldrich (St. Louis, MO, USA).

### 4.3. Application of Exogenous IAA

The application method for IAA followed previous studies, with slight modifications [58]. First, IAA was dissolved in 95% ethanol to prepare a stock solution, which was then diluted with deionized water to the desired concentrations for the working solution. During the V2 vegetative stage, the OX9, OX12, and WT plants were sprayed with 0, 25, and 50 μM concentrations of IAA. After 14 days of application, photographs were taken, and plant height, leaf area, leaf petiole angle, and leaf petiole length were measured. The same experiment was repeated at least twice, and photographs were taken to show representative results.

### 4.4. Transcriptome Analysis

Transcriptome analysis was performed in accordance with a previous study [12]. Here, the analysis process is briefly described as follows: the leaves of WT and OX9 plants were collected from the seedlings in the V2 vegetative stage (day 24 after sowing), and each sample was collected from three biological replications. Total RNA was extracted from each sample. The cDNA was synthesized using adapters and sequenced with the Illumina HiSeq 2500 platform from BGI Genomics (Shenzhen, China). The DEGs of WT and GmMYB14-OX9 line have been further analyzed with fold changes ≥ 2.0 and adjusted *p*-values (q-values) ≤ 0.05.

### 4.5. Quantitative Reverse Transcription PCR (qRT-PCR)

Total RNA of OX12 leaves during the V2 vegetative stage were extracted with Trizol, and then about 1 μg of total RNA was used for reverse transcription using the HiScript 1st Strand cDNA Synthesis Kit (Vazyme, Nanjing, China). The qRT-PCR reactions were run on the CFX Connect^TM^real time PCR System (Bio-Rad, Hercules, CA, USA) using the iTaq^TM^ Universal SYBR Green Supermix (Bio-Rad, Hercules, CA, USA). Reactions were carried out in a 20 μL volume (Bio-Rad iTaq Universal SYBR Green Mix: 10 μL, 10 μM primer F/R: 1 μL, 0.01 μg/μL cDNA: 2 μL, RNase free ddH_2_O: 6 μL), and the cycling program conditions were denaturation at 95 °C for 30 s, followed by 40 cycles of 95 °C for 5 s and 58 °C for 30 s. The soybean *GmActin* gene was used as the reference gene. Data of three biological replicates were analyzed following the relative quantification method (2^−∆∆CT^) [59]. The relative expression levels were normalized to the expression level of Tianlong No.1 (WT). The primers used for RT-qPCR are shown in Table 2.

### 4.6. Electrophoretic Mobility Shift Assay (EMSA)

EMSA was performed essentially as previously described by Hellman et al. [60]. Briefly, the full -length CDS of *GmMYB14* was amplified by PCR and then cloned into the NdeI/XbaI sites of the pCzn1-His expression vector using the ClonExpress II One Step Cloning Kit (Vazyme, Nanjing, China) for the expression of the GmMYB14 protein in *Escherichia coli DE3* strain. The recombinant GmMYB14 protein was purified using Ni-affinity chromatography. EMSA was conducted using an EMSA Kit (Thermo Fisher Scientific, Shanghai, China). DNA-protein complexes were separated on a non-denaturing 6% polyacrylamide gel, transferred to a positively charged nylon membrane, and cross-linked by UV irradiation. Detection of DNA-protein complexes was performed using streptavidin-HRP conjugates, followed by incubation with the substrate from an enhanced chemiluminescence (ECL) kit (Amersham, Buckinghamshire, UK).

### 4.7. Dual-Luciferase Assay

The 1000 bp DNA sequence upstream of the start codon ATG of the *GmIAA1* gene amplified from WT plants and subcloned into the pGreenII-LUC vector to generate the pGmIAA1::LUC reporter plasmid. The empty pGreenII-LUC vector (without promoter insertion) was used as a control. The effector plasmid was constructed by subcloning the full-length CDS of *GmMYB14* into the pAN580 vector under the control of the *35S* promoter. The reporter plasmid (along with the control plasmid) and the effector plasmid were co-transformed into *Arabidopsis* protoplasts, as previously described [61]. Each samples luminescence activity were measuring by the Dual-Luciferase Reporter Assay System e1910 (Promega, Wisconsin). The primers used for the dual-luciferase assay are shown in Table 2.

### 4.8. Data Analysis

Three biological replicates were designed for each experiment. Data are presented as mean values with error bars representing standard deviations (SDs). Student’s *t*-test was employed for significance analysis between two groups, while Duncan’s multiple range test was used for comparisons among multiple groups. All graphs were generated using GraphPad Prism software (version: 8.0.1) [62].

## 5. Conclusions

In summary, our research findings provided new insights into the regulatory mechanisms of dwarfism and compactness architecture of *GmMYB14-OX* lines, suggesting that in addition to the BR pathway, the phenotype of *GmMYB14-OX* plants may be partially attributed to significant reduction of endogenous auxin levels. We also clarified the interaction between GmMYB14 and *GmIAA1* affecting plant growth and development. Our findings have enriched the understanding of the functional diversity of *GmMYB14* and *GmIAA1*, and provide theoretical references for elucidating the regulatory mechanisms of soybean plant architecture.

## Figures and Tables

**Table 1:** Auxin related DEGs of OX9 leaves.

	Class	Gene ID	Fold Changes	*A. thaliana*	Annotations
Up	*GmARF*	*Glyma.14G217700*	4.71	*AT1G19850*	Auxin response factor 5
		*Glyma.10G210600*	3.48	*AT4G30080*	Auxin response factor 10 related
	*GmAUX/IAA*	*Glyma.02G000500*	2.49	*AT5G43700*	Auxin responsive protein IAA 1 related
		*Glyma.05G229300*	3.28	*AT4G29080*	Auxin induced protein
	*GmABP*	*Glyma.15G176900*	3.91	*AT5G20630*	Germin like protein subfamily 3 member 3
		*Glyma.02G150100*	4.58	*AT4G02980*	Auxin binding protein 1
	*GmAUX1/LAX*	*Glyma.02G255800*	4.65	*AT2G38120*	Amino acid transporter
	*GmAXR4*	*Glyma.03G237400*	2.09	*AT1G54990*	Protein auxin response 4 like
Down	*GmARF*	*Glyma.11G145500*	−2.73	*AT2G28350*	Auxin response factor 10 related
		*Glyma.09G072200*	−3.37	*AT5G20730*	Auxin response factor 19 related
		*Glyma.01G002100*	−3.53	*AT5G20730*	Auxin response factor
		*Glyma.13G112600*	−4.41	*AT5G20730*	Auxin response factor 19 related
	*GmAUX/IAA*	*Glyma.02G142500*	−2.23	*AT3G23050*	Auxin responsive protein IAA16
		*Glyma.03G158700*	−2.24	*AT4G14550*	Auxin induced protein AUX28
		*Glyma.20G210500*	−2.14	*AT5G43700*	Auxin responsive protein IAA 1 related
		*Glyma.10G031900*	−2.33	*AT3G23050*	Auxin responsive protein IAA16
	*GmAUX1/LAX*	*Glyma.06G110200*	−2.09	*AT1G77690*	Auxin transporter protein 2 related
		*Glyma.03G063600*	−2.64	*AT2G38120*	Auxin transporter protein 1 related
	*GmPIN*	*Glyma.18G241000*	−3.84	*AT5G16530*	Auxin efflux carrier component 8 related
		*Glyma.07G164600*	−4.12	*AT1G70940*	Auxin efflux carrier component 3 related
	*GmSAUR*	*Glyma.08G004100*	−2.33	*AT2G46690*	Small auxin up RNA
		*Glyma.09G221900*	−3.70	*AT4G38840*	Small auxin up RNA
		*Glyma.09G221800*	−4.78	*AT4G38840*	Small auxin up RNA
		*Glyma.16G020700*	−2.26	*AT2G46690*	Small auxin up RNA
	*GmILR1*	*Glyma.06G115100*	−6.10	*AT1G44350*	IAA amino acid hydrolase ILR1 like 6

**Table 2:** Primers used for qRT-PCR analysis, EMSA, and dual-luciferase assay.

Name	Primer Sequences (5’-3’)	Application
*Glyma.02G255800*-F	TTGACACATTTGGACTCTTGC	qRT-PCR
*Glyma.02G255800*-R	TCAATGATGCAGCTTTGTGTTG	
*Glyma.14G217700*-F	ATGAAGACCACACTTGCAGCAG	
*Glyma.14G217700*-R	TATCAATTTTGCCAAGAGGGTG	
*Glyma.02G150100*-F	GGTAGAACGTTGTACGAGTCCG	
*Glyma.02G150100*-R	AGTCATGTGAGAGAGACCAGCC	
*Glyma.15G176900*-F	GCCTCCAGATTCTGGACTTTTC	
*Glyma.15G176900*-R	TTAACCTGACCCTCCAAGAACA	
*Glyma.10G210600*-F	CACAAGACATCTAATGCTTCCGAT	
*Glyma.10G210600*-R	TTGTCAGTTCCAAGATCAGACTTG	
*Glyam.05G229300*-F	ATGTCTGAGGCATTGGAAGATGT	
*Glyam.05G229300*-R	ATCCACCATTCCAGTTTTCATCA	
*Glyma.03G237400*-F	AGGAAAGTTGAACAGGAGTCCAC	
*Glyma.03G237400*-R	GCGTCCATATAATTAGCTCCATG	
*Glyma.02G255800*-F	TTGACACATTTGGACTCTTTGC	
*Glyma.02G255800*-R	TCAATGATGCAGCTTTGTGTTG	
*GmActin*-F	ATCTTGACTGAGCGTGGTTATTCC	
*GmActin*-R	GCTGGTCCTGGCTGTCTCC	
*GmIAA1*-F	AGCTGTTTAACGGTATGTTTAG	EMSA
*GmIAA*-LUC-F	ACGGTATCGATAAGCTTCGCATTTAAGCTGTTTAACGG	Dual-luciferase assay
*GmIAA*-LUC-R	AGAACTAGTGGATCCGTGACCAGATTATCAGAATCC	

## Data Availability

The original contributions presented in this study are included in the article. Further inquiries can be directed to the corresponding author.
